# Postoperative Radiotherapy for Patients With Resectable Stage III-N2 Non-Small Cell Lung Cancer: A Systematic Review and Meta-Analysis

**DOI:** 10.3389/fonc.2021.680615

**Published:** 2021-07-15

**Authors:** Tianyu Lei, Jing Li, Hao Zhong, Huibo Zhang, Yan Jin, Jie Wu, Lan Li, Bin Xu, Qibin Song, Qinyong Hu

**Affiliations:** Cancer Center, Renmin Hospital of Wuhan University, Wuhan, China

**Keywords:** non-small cell lung cancer, postoperative radiotherapy, surgery, chemotherapy, stage III-N2

## Abstract

**Purpose:**

For resectable cases of stage III-N2 non-small cell lung cancer (NSCLC), the best treatment after surgery is still uncertain. The effect of postoperative radiotherapy (PORT) is controversial. Thus, we performed this updated meta-analysis to reassess the data of PORT in stage III-N2 NSCLC patients, to figure out whether these patients can benefit from PORT.

**Methods:**

We conducted searches of the published literature in EMBASE, PubMed, and the Cochrane Library for relevant randomized control trials (RCTs) comparing PORT group with the non-PORT group in NSCLC patients at stage III-N2. These studies allowed the prior chemotherapy in the treatment. We extracted the data from these articles and used the hazard ratios (HRs) and their 95% confidence intervals (CIs) as summary statistics for estimating the effect of PORT on overall survival (OS), disease-free survival (DFS), local-regional recurrence-free survival (LRFS).

**Result:**

The analyses of seven randomized controlled trials (1,318 participants) show no benefit of PORT on survival (HR, 0.87; 95% CI, 0.71 to 1.07; p = 0.18) but a significantly different effect of PORT on DFS (HR, 0.83; 95% CI, 0.71 to 0.97; p = 0.02) and LRFS (HR, 0.64; 95% CI, 0.50 to 0.81; p = 0.0003). There is not enough evidence of a difference in the effect on survival by the utility of chemotherapy along with PORT though subgroup analysis of no chemotherapy group, concurrent chemoradiotherapy and sequential chemoradiotherapy group. Even in trials with 3D-CRT radiation technique, the pooled analysis shows no benefit of PORT on survival in patients with stage III-N2 NSCLC (data is not shown).

**Conclusion:**

Our findings illustrate that in the postoperative treatment for patients with stage III-N2 NSCLC, PORT contributes to a significantly increased DFS and LR and may not associate with an improved OS, indicating a cautious selection.

## Introduction

Lung cancer is the leading cause of cancer death (18.0% of the total cancer deaths) (1). There are two main forms of lung cancer: NSCLC (85% of patients) and small cell lung cancer (SCLC) (15%) (2). The standard treatment for patients with early stage non-small cell lung cancer (NSCLC) is surgical resection (3), but for patients with apparently completely resected disease, survival is only 40% at five years (4), which may be due to the local-regional recurrent. Especially in patients who are identified as having N2 lymph node involvement, have a worse survival and local-regional recurrence compared with N0 or N1 patients (5). To improve local-regional control of the disease and prolong the survival time, investigators have explored the effect of adjuvant postoperative radiotherapy (PORT) and postoperative chemotherapy (POCT). Burdett et al. (4) initiated an individual participant data meta-analysis for the effect of PORT in NSCLC patients. The pooled analyses showed a significant adverse effect of PORT using cobalt therapy or/and linear accelerators on survival (P = 0.001), with HR 1.18 (95%CI 1.07–1.31). Likewise, data on local-regional recurrence-free survival (HR, 1.12; 95%CI 1.01–1.24) was also significantly in favor of surgery alone without PORT. Many detailed information was included in this trial, while its conclusion could not represent the effect of modern radiotherapy technique. Hence, the role of PORT in NSCLC at stage III-N2 is still unclear. Some previous meta-analyses demonstrated that PORT was associated with improved OS (6, 7), but these meta-analyses included both prospective trials and retrospective studies, which might cause selective bias or other potential bias. Recently, the Lung ART trial in Europe and another trial in China (NCT00880971) have demonstrated different results. Whether patients at stage III-N2 need postoperative radiotherapy or not remains controversial.

Therefore, we include recent high-quality RCTs (evaluated by the ROB2.0 tool provided by the Cochrane website) to perform a meta-analysis to reassess the effect of PORT for resected stage III-N2 NSCLC patients, in an effort to figure out whether patients at stage III-N2 can benefit from PORT. For these patients, chemotherapy is valuable for survival (8, 9), and thus these RCTs allow the prior chemotherapy (pre-operative or post-operative adjuvant chemotherapy, or both) if the research group and the control group both accept the same chemotherapy.

## Materials and Methods

This meta-analysis is based on the Preferred Reporting Items for Systematic Reviews and Meta-analysis (PRISMA) criteria for systematic review and meta-analysis of preferred reporting projects (10) (see **Supplementary Materials**).

### Literature Sources

To identify potentially suitable studies, we searched the Medline, Embase, Cochrane Library, ClinicalTrials.gov for the available published studies before November 6, 2020. We retrieved RCTs from these databases for patients with resectable stage III-N2 NSCLC treated with PORT. The details of the search strategy are presented in **Supplementary Materials**. All published papers with available full texts were retrieved.

### Inclusion and Exclusion Criteria

Studies were included if they met the following criteria: 1) types of participants: completely resected III-N2 NSCLC patients; 2) types of interventions: postoperative radiotherapy ((neo-)adjuvant chemotherapy was allowed); 3) types of outcomes: reported overall survival (OS) or disease-free survival (DFS) or local-regional recurrence survival (LRFS); and 4) types of studies: RCTs only.

If multiple articles covered the same study population, the study with the most recent and complete survival data was utilized. Studies were excluded if any of the following criteria is met: 1) letters, editorials, case reports, reviews and retrospective studies; and 2) survival data could not be extracted from the literature.

**Figure 1:**
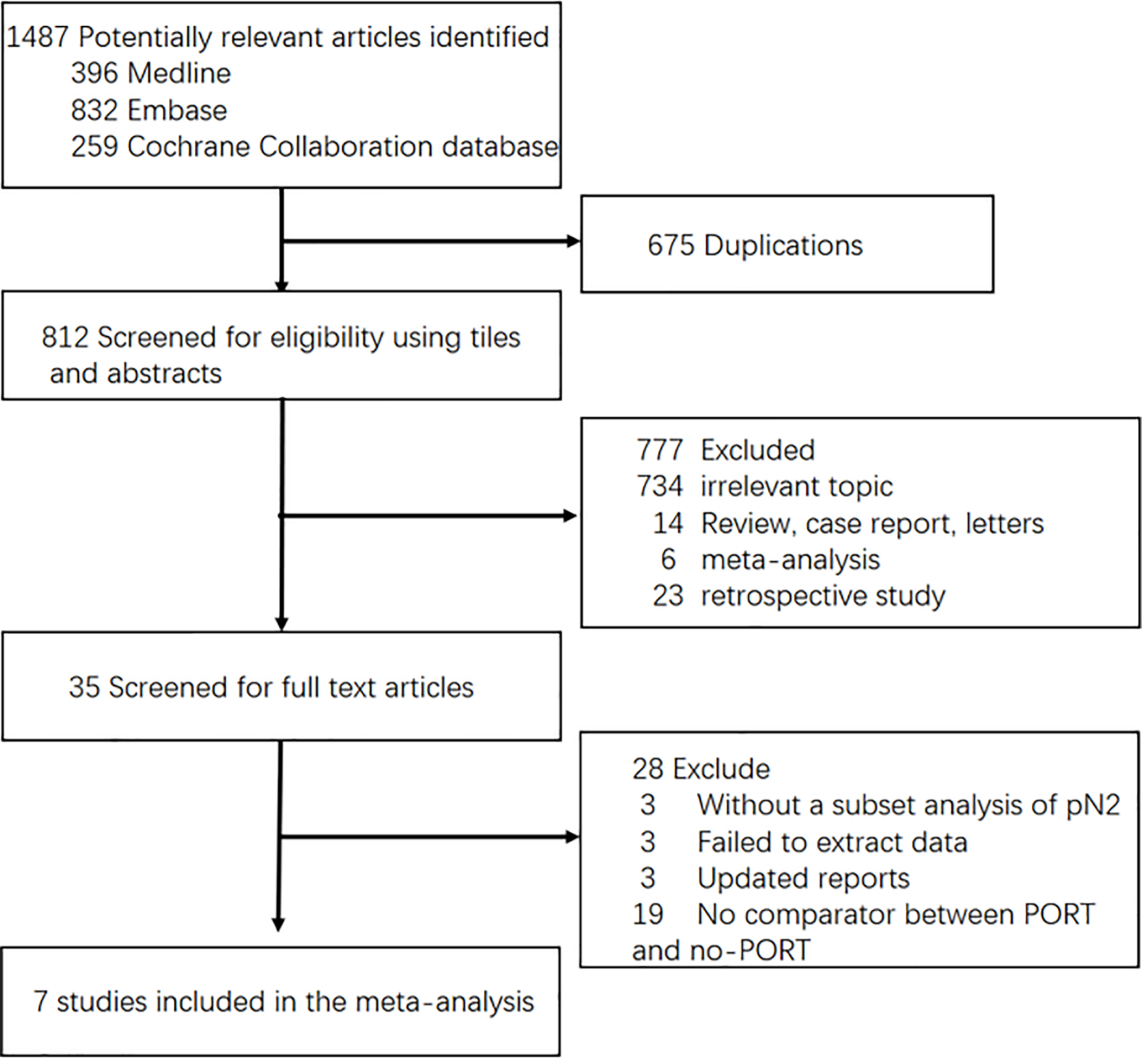
Flow-chart of selecting RCTs for analysis. PORT, Post-operative radiotherapy.

Two investigators checked all the titles and abstracts respectively, and obtained all the full publications for those thought to be potentially relevant. The flowchart is shown in **Figure 1**.

### Data Extractions

The data were extracted by two investigators independently, and the consensus was reached in the case of any discrepancy in all the data. We extracted the following information: first author, years of publication, duration, country of origin, the intervention of each arm, adverse effect, numbers of patient and time-to-event data (OS, DFS or LRFS, especially the value of HR and the 95% confidence interval).

### Quality Assessments

**Figure 2:**
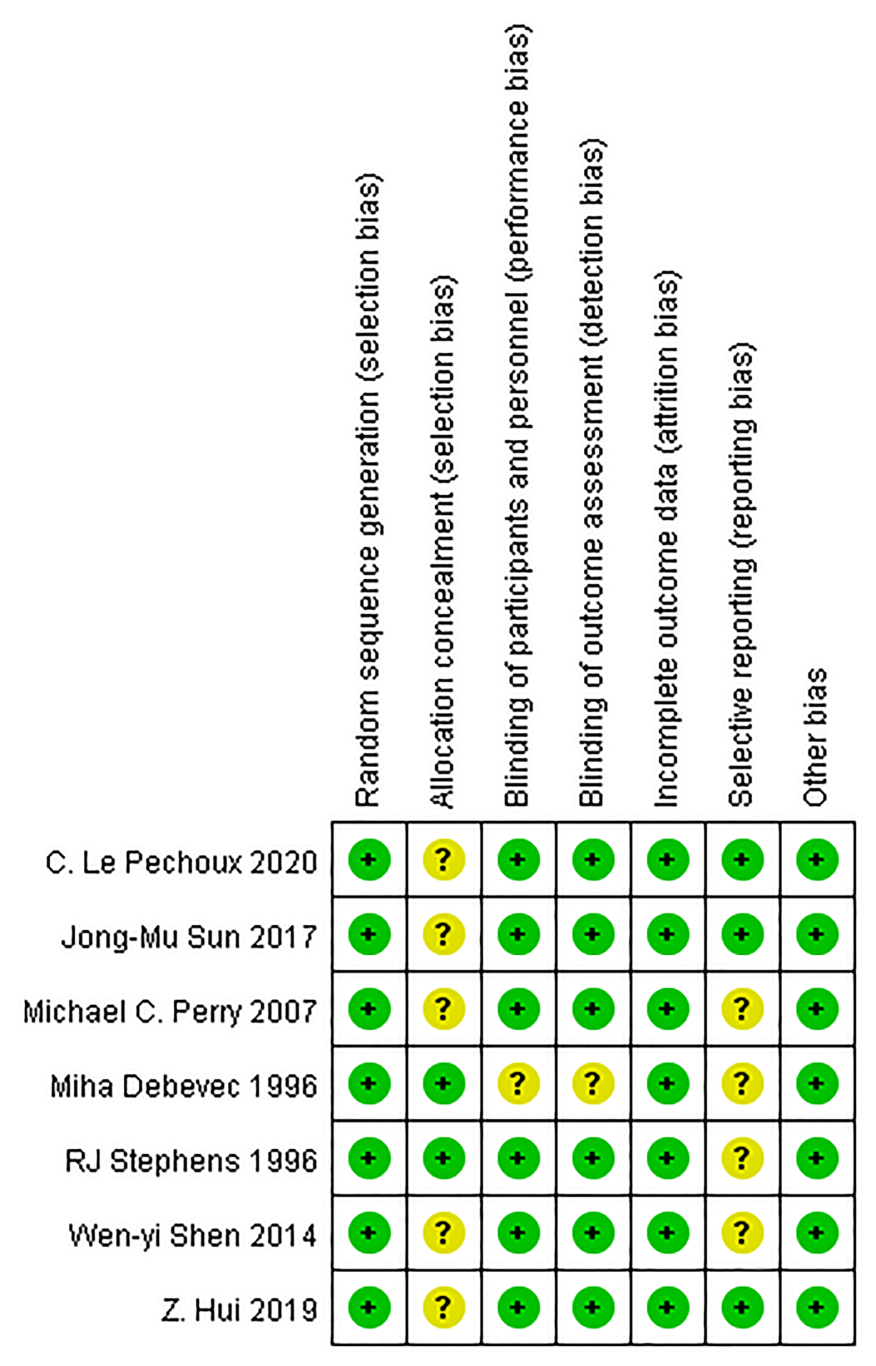
Risk of bias assessment of included studies.

The methodological quality of RCT was assessed by a Cochrane risk of bias tool (11), which was consistent with the following seven domains: 1) random sequence generation; 2) allocation concealment 3) blinding of participants and personnel; 4) blinding of outcome assessment; 5) incomplete outcome data; 6) selective reporting; 7) other bias. The result is shown in the graph of bias risk (**Figure 2**).

### Statistical Analyses

Data were statically analyzed using the software Review Manager 5.3 (Cochrane Collaboration, Oxford, UK). The hazard ratios (HRs) and their 95% confidence intervals (CIs) were used as summary statistics for OS, DFS, and LRFS in the present meta-analysis.

Crude HRs with 95% CIs were extracted directly from the original reports or calculated by the Kaplan–Meier curves or other estimation methods based on the methods supported by Tierney et al. (12). They developed spreadsheet in Microsoft Excel that carries out the calculations for all of the methods described. We used the Engauge-Digitizer (ver11.1) to extract the data from Kaplan–Meier curves. Then the extracted data and estimate censoring using the minimum and maximum follow-up were inputted to the spreadsheet, to obtain similar summary statistics. We made use of the *Chi*-square and *I*-square tests to evaluate the heterogeneity with the significance set at P <0.05 and/or *I*-square >50%. If there is no significant heterogeneity, the fixed effects model will be used, otherwise, the randomized effects model is utilized. The results of the summary HRs are presented in the forest plots. The funnel plot is used to measure the publication bias.

## Results

### Description of Studies

We identified eligible trials and finally included seven trials in this review (see Characteristics of included studies, **Table 1**). We could not include three trials: Smolle-Juettner et al. (20), Mayer et al. (21) and Feng et al. (22). Data for these three trials were unavailable due to the lack of accurate P-value or HR. Thus, this review is based on the results of seven RCTs [Debevec et al. (13); Stephens et al. (14); Perry et al. (15); Shen et al. (16); Sun et al. (17); Hui et al. (18); Pechoux et al. (19)] and 1,318 individuals. Baseline participant characteristics from published literatures show that most participants were male with stage IIIA pN2 squamous cell carcinoma (although histology was unknown for a relatively large number of participants) with good performance status. Among these trials, PORT doses ranged from 30 to 54 Gy, given between 10 and 30 fractions, and considerable diversity was evident in other aspects of radiotherapy planning (**Table 2**). All trials included participants with completely resected tumours for which the disease stage was no greater than IIIB(N2) according to the 8th edition of the AJCC/TNM staging system. Most trials did not provide updated follow-up. In most trials, patients were treated with adjuvant chemotherapy prior or post operation and there were two trials conducting concurrent chemoradiotherapy after resection, two receiving sequential chemoradiotherapy and one having unclear chemotherapy sequence. The other two trials had no chemotherapy.

**Table 1:** Details and results of certain included studies.

Author	Recruitment	Phase of trials	Median age	N	RT technique	Chemotherapy Regimen	Primary end-point	DFS	OS	LRFS
				Patients				HR	HR	HR
Debevec et al. (13)	1988 to 1992	NA	59 (35–80)	35	Linac	without chemotherapy	NA	NA	0.91 (0.44–1.87), NA	NA
				39	–			–	–	–
Stephens et al. (14)	July 1986 to October 1993	NA	62	52	megavoltage x-ray /Cobalt	without chemotherapy	NA	NA	0.74 (0.48–1.15), P = 0.18	0.55 (0.29–1.05), P = 0.07
				54	–			–	–	–
Perry et al. (15)	May 1998 to June 2000	Phase III	NA	19	NA	sequential chemoradiotherapy	NA	NA	0.95 (0.40–2.28), P = 0.91	NA
				18	–			–	–	–
Shen et al. (16)	April 2004 to March 2009	Phase III	NA	66	3DCRT with linac	concurrent chemoradiotherapy	OS and DFS	0.67 (0.45–0.98), P = 0.041	0.69 (0.457–1.044), P = 0.073	HR = 0.48(0.28–0.83), P = 0.009
				69	–			–	–	–
Sun et al. (17)	June 2009 to September 2014	Phase II	60 (38–78)	51	3DCRT with linac	concurrent chemoradiotherapy	DFS	0.94 (0.58–1.52), P = 0.400	1.33 (0.71–2.49), P = 0.38	0.75 (0.36–1.58), NA
				50	–			–	–	–
Hui et al. (18)	January 2009 to December 2017	Phase III	NA	184	3D-CRT/sIMRT	sequential chemoradiotherapy	DFS	0.85 (0.65–1.10), 1-sided P = 0.10	1.01 (0.68–1.51), P = 0.94	0.71 (0.51–0.97), P = 0.03
				180	–			–	–	–
Le Pechoux et al. (19)	August 2007 to July 2018	phase III	61 (36–85)	252	3D-CRT	prior (neo)-adjuvant CT	DFS	0.85 (0.67–1.07), P = 0.16	NA	NA
				249	–			–	–	–

NA, not available.

**Table 2:** The detail of radiotherapy and chemotherapy of included studies.

Trial	Radiotherapy dose				Prescription Technique	Clinical target volume	Chemotherapy
	Total dose (Gy)	Fractions	Durations (weeks)	Gy/day			
Debevec et al. (13)	30	10 or 12	2	2.5 or 3.0	Linac	isolateral hilum and mediastinum	No chemotherapy
Stephens et al. (14)	40	15	3	2.7	megavoltage X-ray and Cobalt	NA	No chemotherapy
Perry et al. (15)	50	25	5	2.0	NA	the mediastinum, supraclavicular fossae, and ipsilateral hilum	Paclitaxel and carboplatin
Shen et al. (16)	50.4	28	6	1.8	3DCRT with linac	ipsilateral mediastinum, hilum and subcarinal lymph node area	paclitaxel and cisplatin
JongMu Sun et al. (17)	50	25	5	2.0	3DCRT with linac	mediastinal lymphatic stations and the immediately adjacent lymph node stations	Adjuvant paclitaxel and carboplatin
Hui et al. (18)	50	25	6	2.0	3D-CRT/sIMRT	Ipsilateral hilum, subcarinal region and ipsilateral mediastinum	platinum based chemotherapy
Le Pechoux et al. (19)	54	27–30	6	1.8–2.0	3D-CRT	NA	prior (neo)-adjuvant CT was allowed

NA, not available.

### Effects of Interventions

Results were finally based on information from seven RCTs (1,318 participants, 659 with PORT, 659 without PORT), representing 99% of individuals from all eligible randomized trials. Overall survival data were available for all trials except Pechoux et al. (19) due to its incompletely published data. Recurrence and disease-free survival data were only available for four trials, respectively. We were not able to get most of the additional information on patients’ characteristics requested from trialists, and thus, some data were not available. Information on age, sex, stage, number of pN2 and histology was not provided for all trials. Performance status data were available for six trials except the trial of Hui et al. (18) and all scored less than 2 expect one patient in surgery group of Debevec et al.’s (13) trials (performance status (Kamofsky) more than 90 = PS (ECOG) 1, 70–90 = 2, less than 70 = more than 2). Therefore, there were insufficient information for the assessment of treatment by covariate interactions.

### Overall Survival

**Figure 3:**
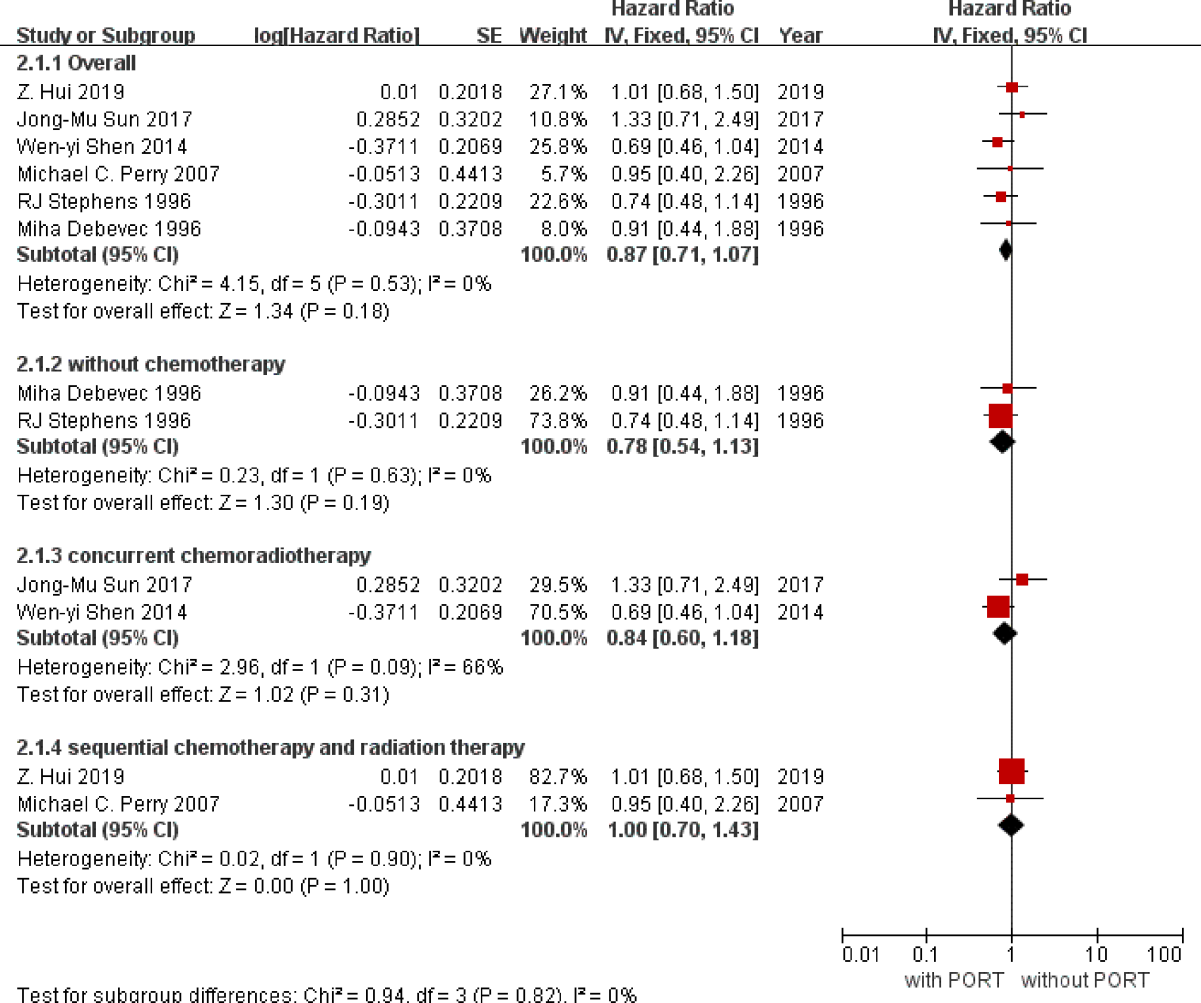
Overall survival. PORT, post-operative radiotherapy; HR, Hazard Ratio.

Overall survival data were available for six trials except the trial of Pechoux et al. (19) with incompletely published data and included information from 817 participants (407 with PORT, 410 without PORT). Although the confidence intervals (CIs) for individual trial results were wide, combined results showed a similar effect of PORT on survival (P = 0.18), with a hazard ratio (HR) of 0.87 (95% CI 0.71 to 1.07) (**Figure 3**). There was no good evidence of increased statistical heterogeneity between trials (*I^2^* = 0%, P = 0.53).

### Disease-Free Survival

**Figure 4:**
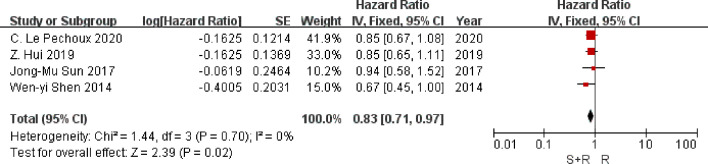
Disease Free Survival. PORT, post-operative radiotherapy; HR, Hazard Ratio.

Data on disease-free survival were available from four trials. Analysis of disease-free survival based on 1,201 patients, gave an HR of 0.83 (95% CI 0.71 to 0.97) in favor of PORT arm (P = 0.02) (**Figure 4**). There was no evidence of gross statistical heterogeneity between trials (*I^2^* = 0%, P = 0.70). Results may indicate a significant decrease in disease-free survival on the PORT arm.

### Local Recurrence-Free Survival

**Figure 5:**
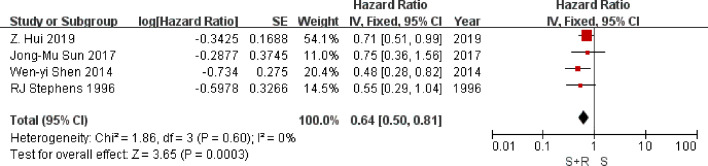
Local-regional recurrence-free survival. PORT, post-operative radiotherapy; HR, Hazard Ratio.

Four trials provided data on local-regional recurrence. Analysis of local-regional recurrence-free survival based on 706 patients, gave an HR of 0.64 (95% CI 0.50 to 0.81), significantly in favor of PORT arm (P = 0.0003) (**Figure 5**). There was no good evidence of statistical heterogeneity between trials (*I^2^* = 0%, P = 0.60), which was consistent with the 1,998 analysis (23) (*I^2^* = 29%, P = 0.19). Results may indicate a significant decrease in local-regional recurrence on the PORT arm.

### Subgroup Analyses

We undertook analyses to assess whether there was any evidence that postoperative radiotherapy had a differential effect in subgroups with or without the order of radiotherapy and chemotherapy. For survival (**Figure 1**), there was no evidence that postoperative radiotherapy was differentially effective in any group of patients defined by without chemotherapy (interaction p = 0.19), concurrent chemoradiotherapy (interaction p = 0.32), or sequential chemoradiotherapy (interaction p = 1.00).

### Sensitivity Analysis and Investigation of Publication Bias

**Figure 6:**
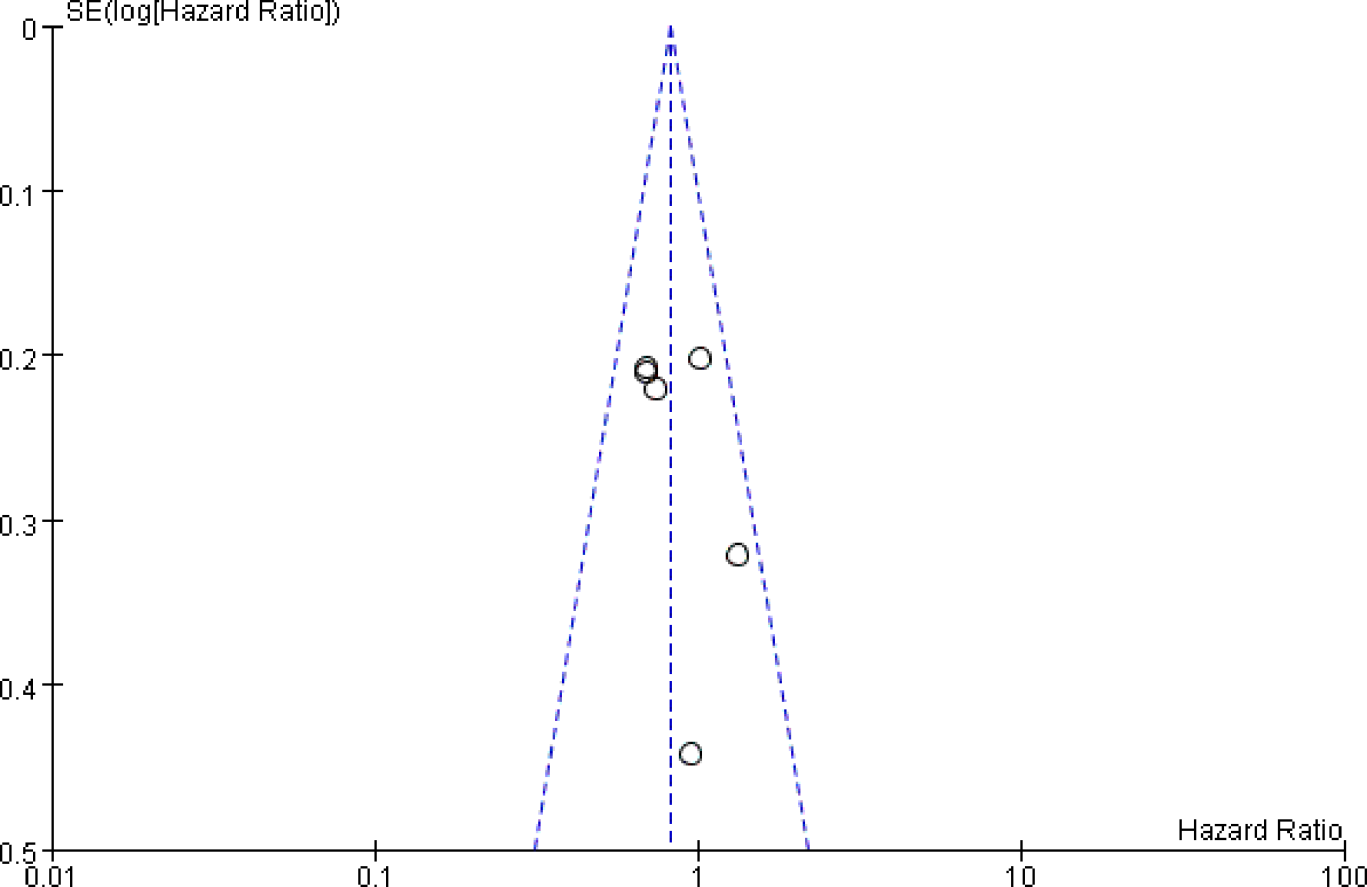
The funnel plot was used to measure the publication bias.

Sensitivity analysis was performed by removing each study sequentially. According to the results, no significant change was observed for pooled HRs, suggesting that all the pooled results were stable and the overall tendency was consistency, indicating no benefit of PORT, which was consistent with previous studies. Publication bias as assessed by Funnel figure (**Figure 6**) indicated no publication bias.

### Toxicity and Side Effect

In the Lung ART trial (19), the PORT group had a higher incidence of grades 3–4 toxicity. According to the cause of death, participants in the PORT group were at a larger risk of dying from the cardio-pulmonary toxicity compared with the control group (16% *vs* 2%), which may partly offset the benefit of local control brought by PORT. In the trial initiated by Sun et al. (17), oral or chest pain was more common in the concurrent chemoradiotherapy arm, while the incidence of myalgia and peripheral neuropathy was higher in the chemotherapy group. The incidence of grades 3–4 toxicity was 36 and 18% in the CCRT arm and chemotherapy, respectively. Another trial (2014) (16) reported that the postoperative concurrent chemoradiotherapy group had a significantly higher incidence of grades 3–4 anemia and esophagitis. Two trials (13, 14) published in 1996 reported that only mild or moderate after effects were observed mainly due to their lower radiation dose compared with the other trials. These evidences suggest that PORT may contribute to a higher incidence of severe toxicity. The improved LRFS and DFS can’t be translated into improved OS may partly attribute to the toxicity.

## Discussion

Due to the question of whether postoperative radiotherapy is effective in the treatment of NSCLC remained unanswered, the clinical trials from different countries and regions are ongoing in spite of varied clinical practice nationally and internationally. The aim of this systematic review and meta-analysis is to provide an updated, reliable and comprehensive summary of favorable effect of postoperative radiotherapy in NSCLC patients, to provide reliable guidance for clinical practice and future research.

For the primary endpoint of survival, there is ambiguity of evidence in the protective role of PORT in NSCLC patients. However, although there is slight tendency that PORT plays a less detrimental role in NSCLC patients compared to 1998 meta-analysis (23) (HR, 1.21, 95% CI 1.08 to 1.34) or 2016 meta-analysis (HR, 1.18, 95% CI 1.07 to 1.31), PORT has no benefit in two recent trials (2017 and 2019). Retrospective studies demonstrated that modern PORT seemed to associate with improved OS compared with no PORT for patients with N2 NSCLC after complete resection and adjuvant chemotherapy (4, 24, 25). Likewise, the difference between concurrent chemoradiotherapy after resection and sequential chemotherapy followed by postoperative radiotherapy was not significant for locally advanced or incompletely resected NSCLC (26), which was consistent with our results. Nevertheless, another clinical trial indicated conducting adjuvant sequential CRT was associated with improved survival over concurrent CRT after completely resection in pN2 NSCLC patients (27). Also, a time to radiation of ≥8 weeks with sequential chemotherapy in the setting of PORT was associated with improved OS (P = 0.0045) in patients with NSCLC with pN2 nodes (28). While another analysis of the National Cancer Data Base reported that for completely resected pN2 NSCLC, adjuvant sequential chemoradiation therapy was associated with improved survival over concurrent chemoradiation therapy (Median OS, 53 months versus 37 months; p <0.001) (27), which might result from the toxicity-related factors. In addition, patients with NSCLC who underwent R0 resection and were found to have pN2 disease had improved outcomes when chemotherapy was administered before radiotherapy compared to concurrent chemotherapy, after propensity score matching (26). In conclusion, adjuvant CT has already been regarded as a standard treatment for patients with pathological diagnosed N2 NSCLC (24). Whereas, it’s a huge question that whether the addition of radiotherapy to PORT with adjuvant chemotherapy in patients with III N2 NSCLC is necessary and rational and the time to use adjuvant PORT is uncertain due to heterogeneity of different studies.

Through reviewing previous researches, we find an analysis based on the SEER Database has reported that PORT was a favorable prognostic factor for patients with stage IIIA N2 disease with ≥6 positive lymph nodes (HR, 0.742; 95% CI, 0.587–0.938; P = 0.012) (29). Likewise, a meta-analysis consisting of 1 randomized controlled trial and 12 retrospective studies suggested that PORT improved both OS [HR, 0.85; 95% CI: 0.79–0.92] and DFS (HR, 0.57; 95% CI: 0.38–0.85) compared with non-PORT treatment in patients with multiple N2 metastases or multiple N2 station involvement, but there was no significant difference in either OS or DFS between PORT and non-PORT groups for patients with single N2 station involvement (30). These evidences suggest that we can screen patients who may potentially benefit from PORT based on the status of the lymph node involvement. Otherwise, two retrospective studies (31, 32) have reported that among patients with stage N2-IIIA NSCLC after surgery, the role of PORT might be related to the pathological type. Patients with lung squamous carcinoma (LUSC) had a poor prognosis than patients with lung adenocarcinoma (LUAD), on the basis of the 5-year OS rates (LUSC 36.3% *vs*. LUAD 41.5%; P = 0.018). However, LUSC patients with limited N2 lymph node metastasis might benefit from PORT compared to postoperative chemotherapy alone (P = 0.010). These two retrospective studies used the propensity score matching analysis to compensate for differences in baseline characteristics, which might improve the reliability of their conclusion.

All analyses of local-regional recurrence-free survival (P = 0.0003), disease-free survival (P = 0.001) have suggested a local protective role of PORT in patient with N2 NSCLC. This conclusion is consistent to the prior studies (33). As for tolerable toxicity, a retrospective research demonstrated that PORT could improve OS, DFS, LRFS and DMFS with tolerable toxicity after pneumonectomy and adjuvant chemotherapy in pIIIA-N2 NSCLC patients (34). In the Lung ART trial (19), the adjuvant PORT brought more adverse events mainly about cardio-pulmonary toxicity with modern 3D-CRT technology. However, the distance metastasis in several trials didn’t show apparent difference between the PORT arm and the control arm. In Lung ART trials, two groups had similar and relatively high system metastasis rates (PORT 72.9% *vs* Control 64.5%), indicating that adjuvant PORT couldn’t improve distant metastasis rates (19). The studies may focus on optimizing treatment regimens to control the metastasis disease. Therefore, whether the utility of immunotherapy or target therapy as emerging systemic treatment agents in patients with pIII-N2 NSCLC will improve their prognosis, it remains to be studied.

Immune checkpoint inhibitors (ICIs), as systematic therapy regent, have been identified to improve survival in patients with advanced NSCLC (35). But for resectable NSCLC, the study is scarce (36). A previous study has largely focused on neoadjuvant immune checkpoint inhibitors. Forde and coworkers showed that single neoadjuvant nivolumab in resectable lung cancer was well-tolerated with few side effects and no delays in surgery (37). Such is the case with sintilimab, another ICI. Likely, neoadjuvant ICIs plus chemotherapy or ICIs regiment was reported to amplify systemic antitumor immunity for achieving a major pathological response, and such that effects could persist after therapeutic surgery (38–41). The WJOG 12119L trial explored the novel treatment strategy of neoadjuvant concurrent chemo-immuno-radiation therapy followed by surgical resection and adjuvant immunotherapy for resectable stage IIIA-B (discrete N2) NSCLC, which might further inhibit distant metastasis during the perioperative period and enhance the prognosis for patients receiving this therapy (42). Notably, atezolizumab is the only effective adjuvant immunotherapy agent following surgery and chemotherapy in people with Stage II-IIIA non-small cell lung cancer (NSCLC) and PD-L1 ≥1%, showing decreasing the risk of disease recurrence or death (disease-free survival; DFS) by 34% (HR, 0.66, 95% CI: 0.50–0.88), compared with best supportive care (BSC) (43). All the trials need to be further validated in large randomized clinical trials.

Additionally, target therapy is recommended to resected pIIIA N2 NSCLC patients with EGFR mutation-positive and receiving prior adjuvant chemotherapy or ineligible to receive platinum-based chemotherapy by the National Comprehensive Cancer Network (NCCN) guidelines. As for resected pIIIA N2 NSCLC patients with EGFR mutation-negative and margin negative (R0), sequential chemotherapy combined with radiotherapy is recommended. Whereas, given that the results of both recent randomized clinic trial Lung ART and this meta-analysis verify that PORT cannot bring benefit for overall survival of these patients, the role of adjuvant PORT is controversial for patients with pIIIA N2 NSCLC. Indeed, currently, although the results of the Lung ART trial (19) were negative, we cannot deny and ignore the value of PORT due to the limitations of this trial. Firstly, the time span of this trial was long, ranging from 2007 to 2018 and in this period, the TNM stage has been revised. Then, the radiotherapy techniques have also been changed to more precise over the times, such as stereotactic body radiation therapy (SBRT) and intensity-modulated radiation therapy (IMRT) while the trial only evaluated the three-dimensional conformal radiation therapy (3D-CRT) with more cardiopulmonary toxicity. In the clinical trial of locally advanced non-small-cell lung cancer, IMRT was considered to correlate with lower rates of severe pneumonitis and cardiac doses compared with 3D-CRT (44). Likewise, a phase II trial has explored that NSCLC patients treated with SBRT had better Health-Related Quality of Life (HRQL) and less toxicity than 3D-CRT (45). With the advancement of radiotherapy technology, from Cobalt-60 to 3D-CRT and then to IMRT or SBRT, radiotherapy technology is gradually getting more precise and reduces the damage to normal tissues. Proton beam therapy (PBT) and carbon ion therapy (CIT) (46, 47), which have emerged in the past decades, can minimize the radiation damage to the human body and have the largest killer effect on tumors due to the Bragg peak effect. In a prospective study (46), the effects of proton radiotherapy and intensity-modulated radiation therapy (IMRT) on postoperative NSCLC were compared, indicating that postoperative PBT in NSCLC is well-tolerated and has similar excellent short-term outcomes when compared with IMRT. However, the study of carbon ion therapy in resectable NSCLC is scarce. In addition to the shift of radiotherapy modality and facilities, the interval between surgery and the onset of radiotherapy and the overall treatment time (OTT) have been reported to be associated with significantly worse local control and overall survival rates (48). Due to the heterogeneity in radiation dose, OTT, fractionation schedules, and the difference between irradiation techniques, the effects of adjuvant PORT are of great difference and heterogeneity.

Furthermore, although trials have been conducted over decades, with changes in diagnosis and assessment of tumour staging, recurrence, and radiotherapy, we still find some consistent conclusion through integrated information including the comparison between different TNM stage versions. This meta-analysis show that no clear evidence indicates the protective effect of PORT on overall survival, but it has a great influence on DFS and LRFS for patients with III-N2 NSCLC. Through the analyses of toxicities and side effects of these randomized clinical trials, we find PORT group may have a higher incidence of severe toxicity compared with no PORT group, which are acceptable and need an early intervention. The improved LRFS and DFS can’t be translated into improved OS may partly attribute to the toxicities. Currently, radiotherapy to the mediastinum after surgery cannot be the standard of care to be recommended for all patients with stage III NSCLC with mediastinal nodal involvement. When adjuvant PORT is recommended to a patient with pIIIA-N2 NSCLC, a comprehensive assessment of the patient’s status is required. Whereas, with the addition of chemotherapy, immune checkpoint inhibitors, and modern and precise radiotherapy techniques and means, this situation may be improved in the future.

### Strengths and Limitations

This meta-analysis analyzed the differential time of added chemotherapy although there was no change of the results which the small sample size might account for. In addition, we added two new published clinical trials specially the Lung ART trial. All the included studies were assessed as having low risk of bias. The inter-study heterogeneity was very low.

The limitations of this analysis are reflected by the fundamental weaknesses of the included trials. Firstly, due to the lack of dada, subgroup analyses could not be performed by patients’ age, sex, histology, and the number of lymph nodes involved, which might influence the extrapolation of the results. Secondly, data are also sparse for survival analysis and cannot draw a Kaplan–Meier curve. Lastly, part of previous trials which may differ from recent studies due to staging, radiotherapy techniques, and chemotherapy influence the reliability of this meta-analysis.

### Implications for Practice and Research

This meta-analysis has shown an ambiguous effect of postoperative radiotherapy on survival in patients with pIII-N2 NSCLC. Although PORT tends to be detrimental in early-stage disease, Researchers must re-evaluate the effect of PORT using modern radiotherapy techniques and adjuvant chemotherapy. A recent systematic review (49) has indicated a benefit effect in OS when PORT is given only with linear accelerators rather than cobalt, cobalt and linear accelerators. Likewise, with the development of modern radiotherapy, including image-guided radiotherapy (IGRT) and intensity modulated radiotherapy (IMRT), contemporary techniques could further decrease PORT-related toxicity, such as the reduced risk of death from heart disease (50).

Meanwhile, adjuvant chemotherapy also plays an important role in the treatment of an N2 NSCLC patient (51) which may change the effect of PORT. In further trials, accurate and detailed information on the cause of death will be important, as will data regarding surgical resection, radiotherapy technique and chemotherapy regimen and sequence. Collection of such data may help to explain whether a combination of adjuvant chemotherapy with surgery contributes to improving benefit effect of postoperative radiotherapy or bring more detrimental effects. At the same time, it helps determine the timing of chemotherapy.

Although, currently, radiotherapy to the mediastinum after surgery cannot be the standard of care to be recommended for all patients with stage III NSCLC with mediastinal nodal involvement, we believe that PORT deserves an in-depth investigation in terms of LR, OS and overall toxicity in patients with resectable stage III-N2 disease especially in patients having received adjuvant systematic therapy such as immune checkpoint inhibitors and target therapy or modern radiation technique to explore novel combined strategies.

## Data Availability Statement

The original contributions presented in the study are included in the article/**Supplementary Material**. Further inquiries can be directed to the corresponding authors.

## Author Contributions

BX and HuZ conceived and designed the study. TL and JL performed the search, study selection, and data extraction and enter. HaZ and LL analyzed the results and the statistical analysis. TL, JL, and YJ drafted the manuscript. Critical review the manuscript and polish the language: HuZ and JW. All authors contributed to the article and approved the submitted version.

## Funding

This work was supported by National Natural Science Foundation of China 81670123 and 81670144. The funders had no role in study design, data collection and analysis, decision to publish, or preparation of the manuscript.

## Conflict of Interest

The authors declare that the research was conducted in the absence of any commercial or financial relationships that could be construed as a potential conflict of interest.
