# Supplementary material for: Postoperative Radiotherapy for Patients With Resectable Stage III-N2 Non-Small Cell Lung Cancer: A Systematic Review and Meta-Analysis
Source: Front Oncol. 2021 Jul 15;11:680615. doi: 10.3389/fonc.2021.680615 (PMC8320322; doi:10.3389/fonc.2021.680615)
Supplement: Supplementary file 1 [file DataSheet_1.docx]

**PICOS**：

**Participant:**

Patients with resectable stage III NSCLC, diagnosis of N2 by histopathology (pIII-N2) according to the AJCC/TNM staging system 8th. The lesions were completely resected without known residual disease.

**Intervention：**

Postoperative radiotherapy with/without ((neo-)adjuvant chemotherapy.

**Comparison:**

Without PORT, the other treatment is consistent with the PORT group.

**Outcome:**

overall survival (OS) or disease-free survival (DFS) or local-regional recurrence survival (LRFS).

**Studies:**

Randomize controlled trials.

# Medline

#1 Search Carcinoma, Non-Small-Cell Lung[MeSH Terms]

#2 Search nsclc[Title/Abstract]

#3 Search lung cancer*[Title/Abstract]

#4 Search lung carcinoma[Title/Abstract]

#5 Search lung neoplasm*[Title/Abstract]

#6 Search lung tumor*[Title/Abstract]

#7 Search lung tumour*[Title/Abstract]

#8 Search non-small cell*[Title/Abstract]

#9 Search nonsmall cell*[Title/Abstract]

#10 Search(( (#1 OR #2) OR #((3 OR #4 OR #5 OR #6 OR #7) AND (#8 OR #9))) AND N2[Title/Abstract])

#11 Search Thoracic surgery[MeSH Terms]

#12 Search surg*[Title/Abstract]

#13 Search thoracic surgical procedures[MeSH Terms]

#14 Search pneumonectomy[MeSH Terms]

#15 Search pneumonectom*[Title/Abstract]

#16 Search lobectom*[Title/Abstract]

#17 Search Lung/surgery[MeSH Terms]

#18 Search thoracotomy[MeSH Terms]

#19 Search Thoracotom*[Title/Abstract]

#20 Search Radiotherapy[MeSH Terms]

#21 Search Radiother*[Title/Abstract]

#22 Search PORT[Title/Abstract]

#23 Search radiation therap*[Title/Abstract]

#24 Search (#11 OR #12 OR #13 OR #14 OR #15 OR #16 OR #17 OR #18 OR #19) AND (#20 OR #21 OR #22 OR #23)

#25 Search #10 AND #24

#26 Search randomized controlled trial[Publication Type]

#27 Search controlled clinical trial[Publication Type]

#28 Search randomized[Title/Abstract]

#29 Search placebo[Title/Abstract]

#30 Search drug therapy[MeSH Terms]

#31 Search randomly[Title/Abstract]

#32 Search trial[Title/Abstract]

#33 Search groups[Title/Abstract]

#34 Search (#26 OR #27 OR #28 OR #29 OR #30OR #31 OR #32 OR #33)

#35 Search animals[MeSH Terms]

#36 Search humans[MeSH Terms]

#37 Search #35 NOT #36

#38 Search #34 NOT #37

#39 Search #25 AND #38

# Embase

#34. #13 AND #32 AND #33

#33. 'randomized controlled trial'/exp OR 'controlled clinical trial'/exp OR randomized:ti,ab OR placebo:ti,ab OR 'drug therapy':lnk OR randomly:ti,ab OR trial:ti,ab OR groups:ti,ab

#32. #30 OR #31

#31. port:ti,ab

#30. #25 AND #29

#29. #26 OR #27 OR #28

#28. 'radiation therap*':ti,ab

#27. radiother*:ti,ab

#26. 'radiotherapy'/exp

#25. #14 OR #15 OR #16 OR #17 OR #18 OR #19 OR #20 OR #21 OR #22 OR #23 OR #24

#24. lobectom*:ti,ab

#23. postoperat*:ti,ab

#22. preoperat*:ti,ab

#21. thoracotom*:ti,ab

#20. 'thoracotomy'/exp

#19. pneumonectom*:ti,ab

#18. 'lung resection'/exp

#17. resect*:ti,ab

#16. surgic*:ti,ab

#15. surger*:ti,ab

#14. 'thorax surgery'/exp

#13. #11 AND #12

#12. 'n2':ti,ab

#11. #1 OR #2 OR #10

#10. (#3 OR #4 OR #5 OR #6 OR #7) AND (#8 OR #9)

#9. 'nonsmall cell*':ti,ab

#8. 'non small cell*':ti,ab

#7. 'lung tumour*':ti,ab

#6. 'lung tumor*':ti,ab

#5. 'lung neoplasm*':ti,ab

#4. 'lung carcinom*':ti,ab

#3. 'lung cancer*':ti,ab

#2. 'nsclc':ti,ab

#1. 'non small cell lung cancer'/exp

# Cochrane central register of controlled trials

#1 lung cancer*

#2 non-small cell*

#3 non small cell*

#4 nonsmall cell*

#5 Mesh descriptor: [Lung Neoplasms] explode all trees

#6 Mesh descriptor: [Carcinoma, Non-Small-Cell Lung] explode all trees

#7 Nsclc

#8 #1 or #2 or #3 or #4 or #5 or #6 or #7

#9 Mesh descriptor: [Thoracic surgery] explode all trees

#10 surg*

#11 Mesh descriptor: [Thoracic Surgical Procedures] explode all trees

#12 Mesh descriptor: [Pneumonectomy] explode all trees

#13 pneumonectom*

#14 lobectom*

#15 Mesh descriptor: [Lung] explode all trees and with qualifier(s):[surgery - SU]

#16 Mesh descriptor: [Thoracotomy] explode all trees

#17 thoracotom*

#18 Mesh descriptor: [Radiotherapy] explode all trees

#19 Radiotherap*

#20 Radiation therap*

#21 PORT

#22 (#9 or #10 or #11 or #12 or #13 or #14 or #15 or #16 or #17) and (#18 or #19 or #20 or #21)

#23 #8 AND N2

#24 #22 AND #23
